# FGFR4 Is Required for Concentric Growth of Cardiac Myocytes during Physiologic Cardiac Hypertrophy

**DOI:** 10.3390/jcdd11100320

**Published:** 2024-10-12

**Authors:** Isaac Campos, Beatrice Richter, Sarah Madison Thomas, Brian Czaya, Christopher Yanucil, Dominik Kentrup, Abul Fajol, Qing Li, Stephen M. Secor, Christian Faul

**Affiliations:** 1 Section of Mineral Metabolism, Division of Nephrology, Department of Medicine, The University of Alabama at Birmingham, Birmingham, AL 35294, USA; icampos6@uab.edu (I.C.); richter.beatrice@yahoo.de (B.R.); smthoma2@uab.edu (S.M.T.); bczaya@mednet.ucla.edu (B.C.); chris@alpha-young.com (C.Y.); dominik.kentrup@northwestern.edu (D.K.); afajol@uabmc.edu (A.F.); qli2@uab.edu (Q.L.); 2 Department of Biological Sciences, University of Alabama, Tuscaloosa, AL 35487, USA; ssecor@ua.edu

**Keywords:** FGF23, FGFR4, physiologic cardiac hypertrophy, pregnancy, python

## Abstract

Fibroblast growth factor (FGF) 23 is a bone-derived hormone that promotes renal phosphate excretion. Serum FGF23 is increased in chronic kidney disease (CKD) and contributes to pathologic cardiac hypertrophy by activating FGF receptor (FGFR) 4 on cardiac myocytes, which might lead to the high cardiovascular mortality in CKD patients. Increases in serum FGF23 levels have also been observed following endurance exercise and in pregnancy, which are scenarios of physiologic cardiac hypertrophy as an adaptive response of the heart to increased demand. To determine whether FGF23/FGFR4 contributes to physiologic cardiac hypertrophy, we studied FGFR4 knockout mice (FGFR4^−/−^) during late pregnancy. In comparison to virgin littermates, pregnant wild-type and FGFR4^−/−^ mice showed increases in serum FGF23 levels and heart weight; however, the elevation in myocyte area observed in pregnant wild-type mice was abrogated in pregnant FGFR4^−/−^ mice. This outcome was supported by treatments of cultured cardiac myocytes with serum from fed Burmese pythons, another model of physiologic hypertrophy, where the co-treatment with an FGFR4-specific inhibitor abrogated the serum-induced increase in cell area. Interestingly, we found that in pregnant mice, the heart, and not the bone, shows elevated FGF23 expression, and that increases in serum FGF23 are not accompanied by changes in phosphate metabolism. Our study suggests that in physiologic cardiac hypertrophy, the heart produces FGF23 that contributes to hypertrophic growth of cardiac myocytes in a paracrine and FGFR4-dependent manner, and that the kidney does not respond to heart-derived FGF23.

## 1. Introduction

Physiologic cardiac hypertrophy is an adaptive response to increased physical demand, such as during pregnancy or consistent exercise, resulting in increased cardiac mass and enhanced cardiac function while maintaining normal morphology [1]. Exercise training provides cardioprotection by improving myocardial tolerance to ischemia–reperfusion injury [2], making this physiological mechanism vitally important to understand for more suitable patient care. Based on the morphologies of the heart and individual cardiac myocytes, cardiac hypertrophy is classified as either concentric or eccentric. Concentric cardiac hypertrophy is characterized by increased heart mass, a reduction in left ventricular chamber dimension, and greater increases in width than in length of individual cardiac myocyte due to a parallel pattern of sarcomere addition [3,4]. Concentric cardiac hypertrophy is primarily pathological, though a milder form can be induced by isometric exercise, such as weightlifting, that is not pathological [4]. Eccentric hypertrophy is characterized by increased heart mass and ventricular volume and by the growth of cardiac myocytes primarily in length due to addition of sarcomeres in series [3]. Non-pathological eccentric hypertrophy is induced by pregnancy and endurance training [5,6]. Unlike pathologic hypertrophy, physiologic hypertrophy is fully reversible [7,8,9], induces angiogenesis [10], and occurs in the absence of interstitial or replacement fibrosis [4] and activation of the molecular stress fetal gene program [11].

Pathologic cardiac hypertrophy is associated with increased levels of endothelin-1 and angiotensin II, leading to activation of their G-protein-coupled receptors [5]. In physiologic hypertrophy, increased levels of growth factors such as insulin-like growth factor (IGF) 1 and vascular endothelial growth factors (VEGF) lead to activation of their corresponding receptor tyrosine kinases on cardiac myocytes [12,13,14,15,16]. Though many studies have investigated the development of physiologic cardiac hypertrophy, the full scope of the inducers and signaling mechanisms involved are still unknown.

Fibroblast growth factor (FGF) 23 is a phosphaturic hormone primarily produced by osteocytes and released into the circulation as an intact protein with a molecular weight of about 32 kDa. FGF23 can also be cleaved between amino acids 179 and 180, leading to the release of N- and C-terminal fragments of 15 and 17 kDa, respectively [17]. Intact FGF23 targets FGF receptor 1 (FGFR1) and the klotho co-receptor in the kidney and the parathyroid gland, thereby activating Ras/mitogen-activated protein kinase (MAPK) signaling, reducing renal phosphate uptake and lowering serum phosphate concentrations. In chronic kidney disease (CKD), serum levels of intact FGF23 are highly elevated and associated with pathologic cardiac remodeling and cardiovascular mortality [18,19], including in dialysis patients [20,21]. Elevated FGF23 is also associated with increased risk of mortality in patients experiencing heart failure with reduced ejection fraction and normal kidney function [22].

We have previously shown that at high concentrations, intact FGF23 can activate FGFR4 on cardiac myocytes, independent of klotho, and initiate phospholipase Cγ (PLCγ)/calcineurin/nuclear factor of activated T cells (NFAT) signaling [23]. FGF23 induces hypertrophic growth of cultured cardiac myocytes via FGFR4, and deletion or blockade of FGFR4 in CKD rodent models with elevated FGF23 protects from cardiac hypertrophy [23]. FGF23 has been shown to induce myocardial fibrosis when overexpressed in the mouse heart via the activation of transforming growth factor beta (TGF-β) and β-catenin [24]. It is likely that FGF23 affects the crosstalk between cardiac myocytes and fibroblasts that leads to pathologic changes in both cell types [25]. Interestingly, FGF23 elevations induced by a high-phosphate diet in the absence of CKD seem to cause cardiac hypertrophy that is reversible [26]. Furthermore, the activation of FGFR4 by the introduction of a *Fgfr4* gain-of-function mutation induces cardiac hypertrophy and the growth of individual myocytes in mice [23]. In contrast, FGF23 elevation in the presence of low phosphate levels, as is the case in X-linked hypophosphatemia (XLH), does not seem to be associated with the development of cardiac hypertrophy in animal models [27,28] or in patients [29,30]. Overall, it appears that FGF23’s actions on the heart are dependent on the context [31]. In the CKD milieu, FGF23 induces cardiac hypertrophy that is accompanied by pathologic alterations, while by itself FGF23/FGFR4 signaling causes hypertrophy that might be beneficial. Previous studies have shown that serum FGF23 levels are elevated in professional cyclists during extreme physical exertion [32] and in pregnant mice [33]. However, whether FGF23 can induce physiologic cardiac hypertrophy is currently unknown.

Since we previously found that FGF23/FGFR4 signaling in cardiac myocytes induces cardiac hypertrophy by activating calcineurin/NFAT signaling [23,34], which has been shown to act as an inducer of pregnancy-associated cardiac hypertrophy [35], we aimed to determine whether FGFR4 is required for the development of physiologic cardiac hypertrophy in pregnant mice and to identify the source of FGF23 in pregnancy. To study FGF23/FGFR4 in a second model of physiologic cardiac hypertrophy, we analyzed Burmese pythons, which experience up to 40% increase in cardiac mass after ingesting a meal [36,37,38].

## 2. Materials and Methods

### 2.1. Antibodies and Recombinant Proteins

We used recombinant murine FGF2 (3139-FB) and FGF23 (2629-FG/CF) proteins from R&D Systems. To inhibit the activity of FGFR4, we used the FGFR4-specific small molecule inhibitor BLU9931 (S7819; Selleckchem, Cologne, Germany).

### 2.2. Mouse Care and Pregnancy Studies

We used three- to four-months old female wild-type C57BL/6J mice purchased from Jackson Laboratories (#000664) and global constitutive FGFR4^−/−^ knockout mice on the C57BL/6 background [39] for the pregnancy study. The females were mated with a proven breeder male mouse. Removing the breeder after 24–48 h or detection of the presence of the copulatory plug was counted as day 1 of pregnancy. Birth most often occurs at days 20–21 of pregnancy. Pregnant mice were studied at day 18 (late pregnancy, LP). Virgin age-matched females (NP) served as controls. On day 18 of pregnancy, mice were weighed and blood was collected via cardiac puncture before euthanasia. The heart, liver, kidneys, and femurs were collected, weighed, and snap-frozen for storage before preparation for further analyses. Isolated hearts were perfused ex vivo (0.5 M KCl in 0.9% NaCl) under pressure (50 mmHg) before further preparation for histological and biochemical analyses. All mice were maintained in temperature-controlled environments with a 12 h light/dark cycle and allowed ad libitum access to food and water.

### 2.3. Serum Chemistry

At endpoint, blood was collected from mice via cardiac puncture, transferred into microvette serum gel tubes (20.1344, Sarstedt, Nümbrecht, Germany) and centrifuged at 10,000× *g* for 5 min, as done before [40]. Serum supernatants were collected and stored at −80 °C. Measurements of serum phosphate and calcium were performed at the Animal Histopathology and Laboratory Medicine Core of The University of North Carolina [10] at Chapel Hill or IDEXX Laboratories Inc. (Buffalo, MO, USA) Serum FGF23 was assessed using ELISAs from Quidel (San Diego, CA, USA) (total FGF23: 60-6300; intact FGF23: 60-6800) with duplicates of serum, diluted 1:2.

### 2.4. RNA Isolation and Quantitative Real-Time PCR

For RNA isolation from snap-frozen mouse tissue, the RNeasy Plus Mini Kit (74136, Qiagen, Hilden, Germany) was used, according to the manufacturer’s protocol. For RNA isolation from long bone, the entire femur was crunched into powder while frozen in liquid nitrogen, and RNA isolation was performed using the RNeasy Lipid Tissue Kit (74804, Qiagen). Crunched bone was incubated with QIAzol lysis reagent (79306, Qiagen) on a rotary shaker at room temperature for 4 h. The dissolved bone was homogenized, incubated for 5 min, and 200 μL chloroform was added. After 3 min of incubation, the mix was centrifuged at 12,000× *g* for 15 min. The upper phase was separated, mixed with 70% ethanol, and applied to the RNeasy column for further RNA isolation. Employing a two-step reaction method, 1 μg of total RNA was reverse-transcribed into cDNA using iScript Reverse Transcription Supermix (1708840, BioRad, Hercules, CA, USA). Quantitative PCR was performed with 100 ng of cDNA, SsoAdvanced Universal SYBR Green Supermix (172-5272, BioRad), and sequence-specific primers (Table 1). Samples were run in triplicate on a CFX96 Touch Real-Time Detection Instrument (1855196, BioRad). Amplification was performed in forty cycles (95 °C, 30 s; 98 °C, 15 s; 60 °C, 30 s; 65 °C, 5 s). The generated amplicon was systematically double-checked by its melting curve. Relative gene expression was normalized to expression levels of housekeeping gene *Gapdh*. Results were evaluated using the 2^−∆∆Ct^ method and expressed as mean ± standard deviation (SD).

### 2.5. Histology and Morphometry of Mouse Hearts

To prevent bias in the measurement of myocyte cross-sectional area using paraffin-embedded transverse cardiac sections, researchers taking images and measuring cell area were blinded. Perfused heart tissue meant for histology was additionally washed with cold saline ex vivo and fixed overnight in 4% phosphate-buffered formaldehyde solution. Tissue was sent to IDEXX Bioanalytics (Columbia, MO, USA) for embedding, sectioning, and staining with hematoxylin and eosin (H&E) or Masson’s trichrome. Transverse sections stained with H&E, Masson’s trichrome, or following wheat germ agglutinin (WGA) staining were imaged on a Leica DMi8 microscope (Berlin, Germany).

Short-axis heart sections stained with H&E were used to quantify myocardial thickness by measuring the distance from the inner to the outer myocardial edges at the mid-chamber zone. Mean left ventricular free-wall thickness was calculated from seven measurements of wall thickness taken at 0, 30, 60, 90, 120, 150, and 180 degrees along the hemi-circle of the short axis of the free wall. Mean septum thickness was calculated from seven measurements of wall thickness taken at 0, 30, 60, 90, 120, 150, and 180 degrees along the hemi-circle of the short axis of the septum. Images of the entire heart sections were taken using 20× magnification and the scanning imaging capabilities of the Leica DMi8 microscope.

Short-axis heart sections stained with Masson’s trichrome were used to quantify the connective tissue fraction. Images of the entire heart sections were taken using 20× magnification and the scanning imaging capabilities of the Leica DMi8 microscope. Images of the entire heart section were taken using the same parameters for each image, then analyzed using ImageJ software v1.54j (NIH, Bethesda, MD, USA) and the publicly available “*Colour Deconvolution2*” plug-in to “un-mix” the collagen fiber staining from others. Analyses of these sections were performed using the same threshold parameters for each image [41]. The data from 2 sections per heart were averaged to generate an n = 1.

For WGA staining, paraffin sections underwent deparaffinization twice for 5 min in Shandon Xylene Substitute and then rehydrated through a graded ethanol series (99%, 97%, 70%), twice for 5 min each. Antigen retrieval was performed in a microwave for 15 min in unmasking solution (H3300; Vector Labs, Newark, CA, USA). Slides were washed 3 times for 5 min each in PBS (BP243820; Thermo Fisher, Waltham, MA, USA), then incubated for 1 h in blocking solution [1% BSA (BSA-50; Rockland, Colombo, Sri Lanka), 0.1% cold water fish skin gelatin (900033; Aurion, Toowong, Australia), and 0.1% Tween 20 (P1379; Sigma-Aldrich, Saint Louis, MO, USA)]. Slides were washed 3 times in PBS and then incubated in 10 μg/mL of 594-conjugate WGA (W11262; Thermo Fisher) for 1 h. Slides were washed 3 times with PBS and then mounted in Prolong Diamond (P36961, Thermo Fisher). Immunofluorescence images were taken with a 63× oil objective on a Leica DMi8 microscope. ImageJ software (NIH) was used to quantify the cross-section area of 25 myocytes per field in 4 fields along the mid-chamber free wall based on WGA-positive staining.

### 2.6. Snake Care and Sample Acquisition

Captive-born hatchling Burmese pythons (*Python bivittatus*) were purchased commercially and housed individually in 20 L plastic containers at 28–32 °C under a 14 h:10 h light:dark cycle. Pythons were fed laboratory rats every 2 weeks and had continuous access to water. Seven-week-old CD-strain rats were purchased frozen (RodentPro, Evansville, Indiana) and thawed in clean warm water before feeding. Prior to experimentation, food was withheld from snakes for a minimum of 30 days to ensure that the snakes were postabsorptive. Burmese pythons are known to complete their digestion within 10–14 days after feeding [38]. Snakes used in this study were of both sexes and were between 12 and 36 months old. To induce the postprandial response, pythons were fed rodent meals equivalent to 25% of their body mass.

Diamondback water snakes (*Nerodia rhombifer*) were captured by hand from commercial catfish ponds in Leflore County, MS, USA. At these ponds, water snakes have continuous access to food (channel catfish, *Ictalurus punctatus*) and were frequently observed feeding. Water snakes were maintained in a large 3000 L tank, under a 14 h:10 h light:dark cycle, at 25–28 °C. Both sexes were used for this study. Water snakes were fed catfish fillets weekly and had continuous access to water. Laboratory studies have found that their stomachs are cleared 4–5 days after feeding [42]. Water snakes were analyzed after being fasted for 15 days or at 2 days following the consumption of catfish fillet meals that weighed 25.1 ± 0.1% of snake’s body mass. Mean body mass of water snakes did not differ between the fasted and fed groups.

Prior to the collection of tissues and serum, snakes were euthanized by severing the spinal cord immediately posterior to the head. Serum was collected from fasted snakes and snakes 12 h post-feeding (HPF), 2 days post-feeding (DPF), and 3 DPF, as done before [40]. Samples were snap-frozen in liquid nitrogen and stored at −80 °C prior to experimentation.

### 2.7. Isolation and Culture of Neonatal Rat Ventricular Myocytes

Neonatal rat ventricular myocytes (NRVM) were isolated using a standard cell isolation system from Worthington Biochemical Corporation (LK003300), detailed in [34]. Hearts from 1–2 days-old Sprague Dawley rats were harvested, minced in calcium- and magnesium-free Hank’s Balanced Salt Solution (HBSS) from the kit, and the tissue digested with 50 μg/mL trypsin at 4 °C for 16–20 h. Soybean trypsin inhibitor in HBSS was added, and the tissue was further digested with collagenase in Leibovitz L-15 medium (11415064; Thermo Fisher) under slow rotation (15 rpm) at 37 °C for 1 h. Cells were released by triturating the suspension 20 times with a standard 10 mL plastic serological pipette and filtering it through a cell strainer [70 μm, BD Falcon (08-771-2; Fisher Scientific)]. Cells were incubated at room temperature for 20 min and spun at 50 g for 5 min. After resuspension in plating medium [Dulbecco’s Modified Eagle Medium; DMEM (10-013-CV; Corning, NY, USA) with 17% Medium 199 (11150059; Gibco, Waltham, MA USA), 15% FBS (16000044; Gibco), and 1% penicillin/streptomycin solution [P/S (15140122; Thermo Fisher)], cells were counted using a hemocytometer. Then, 3 × 10^5^ cells were seeded on laminin (23017015; Gibco)-coated [10 μg/mL in PBS] glass cover slips in 24-well plates. Cells were left undisturbed in plating medium at 37 °C for 72 h and then cultured in maintenance medium [DMEM with 20% Media 199, 1% insulin–transferrin–sodium selenite solution [ITS (I3146; Sigma-Aldrich)] and 1% P/S] in the presence of 100 μM 5-bromo-2′-deoxyuridine [BrdU (B5002; Sigma-Aldrich)] for 4 more days. Isolated cardiac myocytes were then treated for 48 h in BrdU-containing maintenance medium in the presence of recombinant FGF2 (25 ng/mL) or heat-inactivated serum (at final concentration of 2%) from fasted pythons, pythons 12HPF, pythons 3DPF, fasted water snakes, and water snakes 2DPF. These treatments were done with and without the 1 h pre-treatment with FGFR4 inhibitor (BLU9931; 10 ng/mL). For each treatment of NRVMs, serum came from a different snake within the corresponding group.

### 2.8. Immunohistochemistry and Morphometry of NRVMs

Cultured NRVMs were fixed using 2% paraformaldehyde (PFA) in 5 mg/mL sucrose for 5 minutes and permeabilized in 0.3% Triton-X100 (Sigma-Aldrich) in PBS for 10 min. NRVM were stained for sarcomeric α-actinin [1:1000 (EA-53; Sigma-Aldrich)]. Cy3-conjugated goat-anti-mouse (115-165-003; Jackson Immuno Research, West Grove, PA, USA) was used as secondary antibody at 1:300. Nuclei were visualized with 4′,6-diamidino-2-phenylindole [DAPI (D1306; Fisher Scientific) 400 ng/mL in PBS for 10 minutes]. Immunofluorescence images were taken on a Leica DMi8 microscope with a 63× oil objective. ImageJ software (NIH) was used to quantify myocyte cross-sectional area based on α-actinin-positive staining. One hundred myocytes were measured and averaged per slide; three slides were analyzed per treatment condition.

### 2.9. Statistics

**Figure 1:**
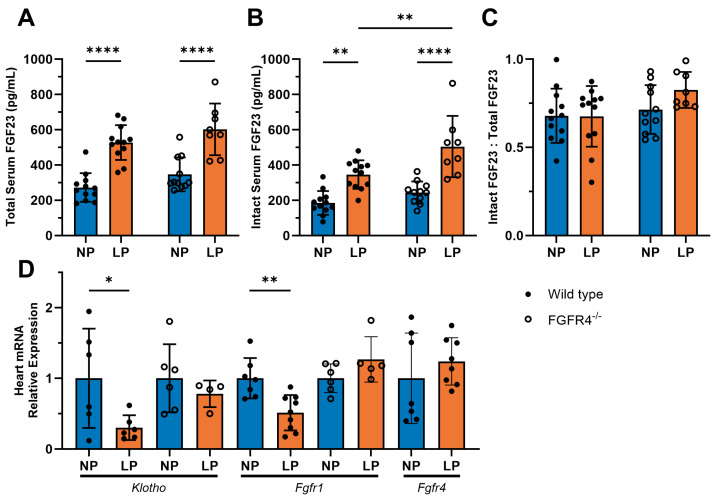
Serum FGF23 levels are elevated in mice during late pregnancy. Wild-type and global FGFR4 knockout (FGFR4^−/−^) mice were investigated during late pregnancy (LP; 18 days post-mating) and compared to non-pregnant virgin littermates (NP). Serum FGF23 concentrations were measured via ELISA. Both (**A**) total FGF23 and (**B**) intact FGF23 were significantly elevated in the serum of wild-type (total **** *p* < 0.0001; intact ** *p* = 0.0014) and FGFR4^−/−^ mice (total **** *p* < 0.0001; intact **** *p* < 0.0001) when compared to their respective NP controls (n = 8–12 mice per genotype and timepoint). FGFR4^−/−^ LP mice exhibit significantly higher intact FGF23 compared to wild-type LP mice (** *p* = 0.0055). (**C**) The ratios of intact FGF23 over total FGF23 show no significant differences between groups (n = 8–12 mice per genotype and timepoint). (**D**) Cardiac tissue from LP mice showed significant decreases in *Klotho* expression in wild-type mice (* *p* = 0.0398) compared to wild-type NP mice, but there were no significant differences between NP and LP in FGFR4^−/−^ mice (n = 4–6 mice per genotype and timepoint). Cardiac tissue from wild-type mice in LP showed a significant decline in *Fgfr1* mRNA levels (** *p* = 0.0026), while FGFR4^−/−^ mice had no significant change in expression when compared to their respective NP control mice (n = 6–9 mice per genotype and timepoint). *Fgfr4* expression in cardiac tissue from wild-type mice in LP remained unaltered, compared to wild-type mice in LP (n = 7–8 mice per genotype and timepoint). RTqPCR analysis of tissue was conducted using *Gapdh* as a housekeeping gene. All values are shown as ±SD.

**Figure 2:**
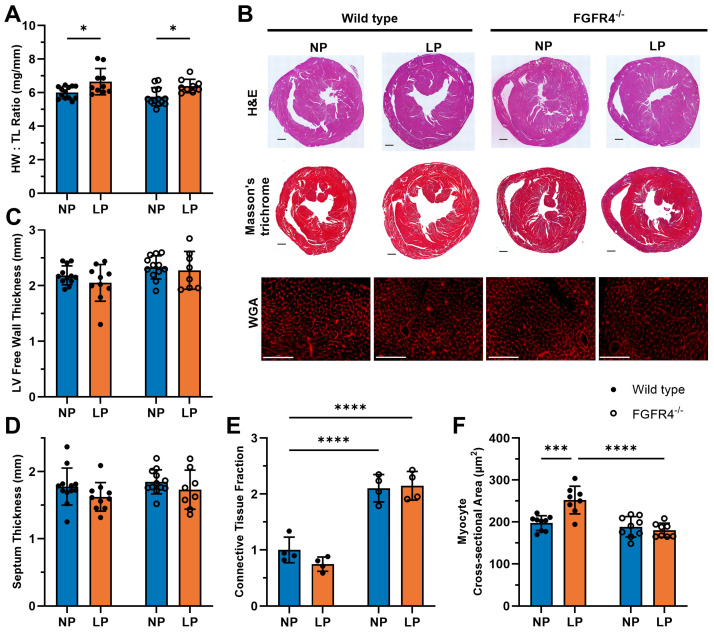
FGFR4 deletion prevents pregnancy-related concentric growth of individual cardiac myocytes. Wild-type and FGFR4 knockout (FGFR4^−/−^) mice in late pregnancy (LP; 18 days post-mating) exhibited increased (**A**) heart weight/tibia length ratios when compared to their respective non-pregnant (NP) controls (wild-type * *p* = 0.0322; FGFR4^−/−^ * *p* = 0.0397; n = 9–12 mice per genotype and timepoint). (**B**) Representative gross pathology of mid-chamber sections of the heart with H&E staining (original magnification, 20×; scale bar: 200 μm), Masson’s trichrome staining of heart mid-chamber sections (original magnification, 20×; scale bar: 200 μm), and left ventricular mid-chamber sections stained with wheat germ agglutinin (WGA; original magnification, 40×; scale bar: 100 μm). Compared to NP, mice in LP did not have altered (**C**) left ventricular free-wall thickness or (**D**) interventricular septum thickness regardless of the genotype (n = 8–12 mice per timepoint and genotype). (**E**) The connective tissue fraction, determined by Masson’s trichrome staining, in FGFR4^−/−^ mice was significantly greater, regardless of pregnancy condition, when compared to wild-type NP mice (NP **** *p* < 0.0001; LP **** *p* < 0.0001; n = 4 mice per genotype and timepoint; each n is the average of 2 cross-sections per heart). (**F**) The cross-sectional area of individual myocytes was significantly increased in wild-type mice during LP, compared to NP controls (*** *p* = 0.0002) and FGFR4^−/−^ LP mice (**** *p* < 0.0001; n = 9–10 mice per genotype and timepoint; 100 cells averaged per mouse). Alterations in individual myocyte area are not observed in FGFR4^−/−^ mice at LP (n = 8–9 mice per genotype and timepoint; 100 cells averaged per mouse). All values are shown as ±SD.

**Figure 3:**
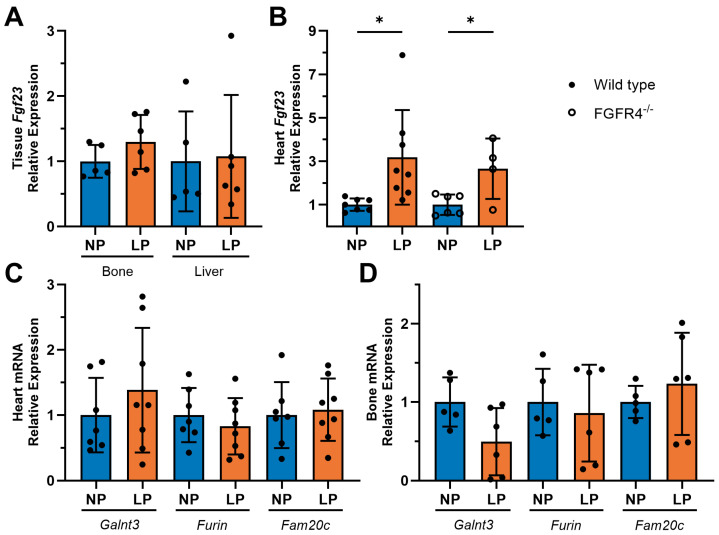
FGF23 is expressed in the heart during late pregnancy in mice. (**A**) In wild-type mice during late pregnancy (LP; 18 days post-mating), *Fgf23* mRNA levels were not altered in bone or liver tissue compared to non-pregnancy (NP) (n = 5–6 mice per timepoint). (**B**) Cardiac tissue from LP mice showed significant increases in *Fgf23* expression in both wild-type (* p = 0.0213) and FGFR4 knockout (FGFR4^−/−^) (* *p* = 0.0246) mice when compared to respective NP controls (n = 4–8 mice per genotype and timepoint). (**C**) In cardiac tissue of wild-type mice, no differences in the mRNA levels of the FGF23 regulatory genes *Galnt3*, *Furin*, or *Fam20c* were observed between NP and LP (n = 7–8 mice per timepoint). (**D**) Bone tissue from wild-type mice showed no differences in mRNA levels of *Galnt3*, *Furin*, or *Fam20c* (n = 5–6 mice per timepoint) in NP versus LP. RTqPCR analysis of tissue was conducted using *Gapdh* as a housekeeping gene. All values are shown as mean ± SD.

**Figure 4:**
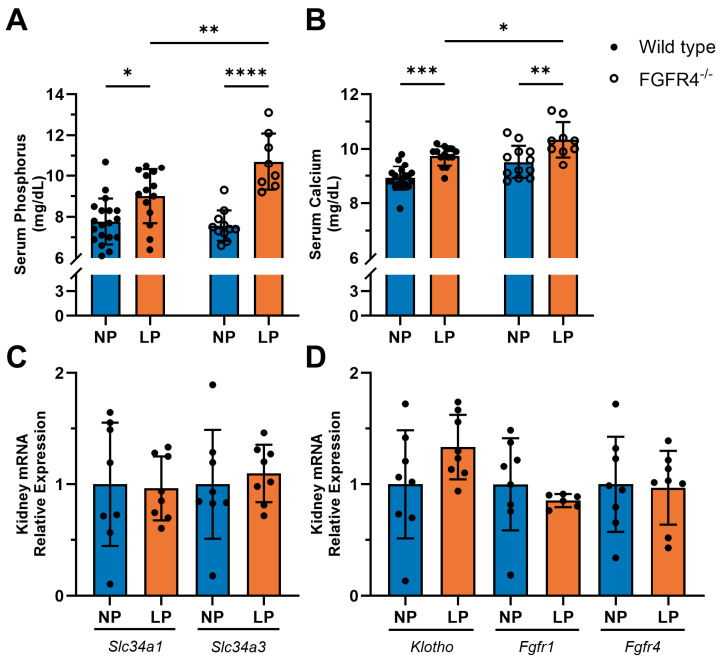
Pregnant mice have increased serum phosphorus and calcium despite FGF23 elevations. (**A**) Serum phosphorus was significantly elevated during late pregnancy (LP; 18 days post-mating) in wild-type mice (* *p* = 0.0196; n = 14–19 mice per timepoint) and FGFR4 knockout (FGFR4^−/−^) mice (**** *p* < 0.0001; n = 8–11) when compared to non-pregnant (NP) mice. FGFR4^−/−^ LP mice have significantly higher serum phosphorus compared to wild-type LP mice (** *p* = 0.0093; n = 8–14). (**B**) Serum calcium was also significantly elevated in pregnant wild-type (*** *p* = 0.0001; n = 14–19) and FGFR4^−/−^ mice (** *p* = 0.0026; n = 9–12), compared to NP controls. FGFR4^−/−^ LP mice have significantly higher serum calcium compared to wild-type LP mice (* *p* = 0.0364; n = 9–14). Renal tissue from wild-type mice in LP exhibited no alterations in the mRNA levels of (**C**) sodium-dependent phosphate transporters *Slc34a1* and *Slc34a3* or of (**D**) *Klotho*, *Fgfr1*, or *Fgfr4* (n = 8 mice per timepoint). RTqPCR analysis of tissue was conducted using *Gapdh* as a housekeeping gene. All values are shown as mean ± SD.

**Figure 5:**
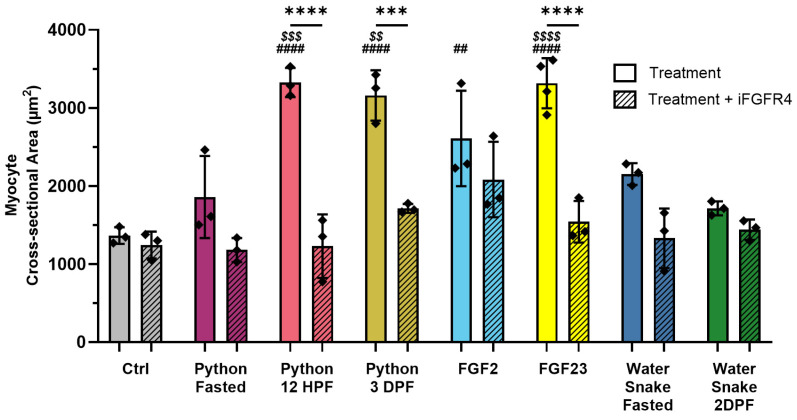
FGFR4 inhibition abrogates cardiac myocyte hypertrophy induced by serum from fed pythons. Neonatal rat ventricular myocytes (NRVMs) were treated with media containing either vehicle, FGF2 (25 ng/mL), FGF23 (25 ng/mL), or serum from fasted or fed snakes (diluted to 2% of the total media volume), without or in combination with a small molecule inhibitor of FGFR4 (iFGFR4; BLU9931, 10 ng/mL), for 48 h. Compared to vehicle-treated NRVMs (Ctrl) or to NRVMs treated with serum from fasted pythons, serum collected from pythons twelve hours post-feeding (Python 12HPF; ^####^ *p* < 0.0001) and three days post-feeding (Python 3DPF; ^####^ *p* < 0.0001) induced a significant increase in myocyte area. When co-treated with iFGFR4 (textured bars), both Python 12HPF (**** *p* < 0.0001) and Python 3DPF (*** *p* = 0.0003) did not induce a significant increase in NRVM area when compared to the same treatment without iFGFR4. Python 12HPF (^$$$^ *p* = 0.0003)- and Python 3DPF (^$$^ *p* = 0.0016)-treated NRVMs had significantly increased myocyte area when compared to Python Fasted serum treatments. Treatment with recombinant FGF23 significantly increased myocyte area compared to Ctrl (^####^ *p* < 0.0001), fasted python serum treatments (^$$$$^ *p* < 0.0001), and FGF23 + iFGFR4 treatment (**** *p* < 0.0001) NRVMs. Treatment with recombinant FGF2 protein served as a positive control for hypertrophy (^##^ *p* = 0.003). Treatment with serum from fasted and two-days-post-feeding water snakes (Water Snake Fasted, Water Snake 2DPF) served as negative controls Each individual color corresponds with the treatment indicated below the bars. # = versus Ctrl; * = versus same treatment + iFGFR4; $ = versus Python Fasted; 150 cells per condition; n = 3–4 independent isolations of NRVMs. All values are shown as mean ± SD.

Data organization, scientific graphing, and statistical significance of differences between experimental groups were performed using GraphPad Prism (version 10.3.1). All results are expressed as mean ± SD. Comparisons between three or more groups on 2 different categorical variables were performed by 2-way ANOVA with post hoc Tukey’s multiple comparison test; this was used for all data except for mRNA expression analyses. For all data in Figure 1, Figure 2, Figure 3 and Figure 4, the two categorical variables investigated were pregnancy condition (NP vs. LP) and genotype (wild-type vs. FGFR4^−/−^), with pregnancy condition consistently being the main effect. In Figure 5, the two categorical variables investigated were treatment of NRVMs and the presence vs. absence of FGFR4 inhibitor (Treatment vs. Treatment + iFGFR4). In Figure 5, both factors had equally significant effects (*p* < 0.0001). Comparisons between two groups were performed by two-tailed *t*-tests and were used to analyze the gene expression within an individual genotype between NP and LP groups. A significance level of *p* ≤ 0.05 was accepted as statistically significant. Sample size was determined on the basis of sample availability, prior experimental studies performed in our laboratory, and from prior literature. No formal randomization was used in any experiment. For in vivo experiments, animals were unbiasedly assigned into different experimental groups, regardless of genotype. Group allocation was not performed in a blinded manner. Whenever possible, investigators were blinded to experimental groups (for example, in IF and IHC experiments, by hiding group designation and genotype of animals until after quantification and analysis). 

## 3. Results

### 3.1. Pregnant Mice Develop Elevated Serum FGF23 Levels and Require FGFR4 for the Hypertrophic Growth of Cardiac Myocytes

To determine whether serum FGF23 levels are elevated during pregnancy, we studied wild-type mice and mice with global deletion of *Fgfr4* (FGFR4^−/−^) in late pregnancy (LP; 18 days post-mating) and compared them to non-pregnant virgin-littermate controls (NP). Regardless of the genotype, LP mice had significant increases in serum concentrations of total (wild-type *p* < 0.0001; FGFR4^−/−^ *p* < 0.0001) and intact FGF23 (wild-type *p* = 0.0014; FGFR4^−/−^ *p* < 0.0001), compared to NP controls (Figure 1A,B). Wild-type and FGFR4^−/−^ mice in LP showed similar elevations in serum levels of total FGF23 (Figure 1A). Interestingly, FGFR4^−/−^ mice in LP developed significantly (*p* = 0.0055) higher elevations of intact FGF23 compared to the concentrations observed in wild-type mice during LP (Figure 1B). We also calculated the ratio of intact FGF23 to total FGF23 and found that there was no significant difference between groups or genotypes (Figure 1C). When we analyzed isolated heart tissue for the expression of potential FGF23 receptors by RTqPCR, we found that in LP, cardiac *Klotho* expression was significantly decreased in wild-type mice (*p* = 0.0398) but not in FGFR4^−/−^ mice when compared to their respective NP controls (Figure 1D). *Fgfr1* was significantly decreased in wild-type LP mice when compared to NP mice (*p* = 0.0026), while FGFR4^−/−^ LP mice did not show significant changes in *Fgfr1* expression when compared to their respective NP controls (Figure 1D). Furthermore, cardiac *Fgfr4* levels in wild-type mice were not significantly different between NP and LP (Figure 1D). These findings indicate that in wild-type mice, LP is accompanied by increases in serum FGF23 levels and by decreases in the cardiac expression of FGFR1 and Klotho. Moreover, the absence of FGFR4 does not alter the effects of LP on circulating FGF23 concentrations.

To determine the potential role of FGF23/FGFR4 in pregnancy-associated cardiac hypertrophy, we conducted histological analyses of hearts from wild-type and FGFR4^−/−^ mice. In mice in LP, we observed significant (wild-type *p* = 0.0322; FGFR4^−/−^ *p* = 0.0397) increases in heart-weight-to-tibia-length ratios in both genotypes, when compared to their respective NP controls (Figure 2A). Histological analyses of H&E-stained cardiac sections (Figure 2B) demonstrated that wild-type and FGFR4^−/−^ mice did not exhibit increased left ventricular (LV) wall (Figure 2C) or interventricular septum (Figure 2D) thickness in LP, compared to NP mice. Histological analysis of Masson’s trichrome-stained cardiac sections (Figure 2B) showed that FGFR4^−/−^ mice have significantly increased connective tissue fraction (NP *p* < 0.0001; LP *p* < 0.0001) when compared to wild-type NP mice (Figure 2E). This difference between wild-type and FGFR4^−/−^ mice was observed in both NP and LP, indicating that the lack of global FGFR4 signaling or specifically cardiac FGFR4 during the development of the heart affects the cardiac connective tissue. The analysis of WGA staining of heart sections (Figure 2B) showed that wild-type mice have increased cardiac myocyte cross-sectional area in LP compared to NP wild-type (*p* = 0.0002) and to LP FGFR4^−/−^ (*p* < 0.0001) mice. FGFR4^−/−^ mice did not experience elevations in the area of individual cardiac myocyte during LP, compared to NP FGFR4^−/−^ controls (Figure 2F). These data indicate that in mice during LP, when serum and cardiac FGF23 levels are elevated, FGFR4 is required for the development of cardiac hypertrophy on the cellular level.

### 3.2. In Pregnant Mice, FGF23 Is Produced by the Heart, but Renal Expression of Genes Related to Phosphate Homeostasis Is Unaltered

Since we detected increases in serum FGF23 levels during pregnancy, we wanted to identify the source of FGF23. Interestingly, the RTqPCR analysis of wild-type mice showed no differences in *Fgf23* expression between NP and LP in bone (Figure 3A). Similarly, LP did not affect *Fgf23* expression in the liver. However, when we analyzed isolated heart tissue, we found that wild-type mice had significantly increased *Fgf23* expression in LP when compared to NP controls (*p* = 0.0213). This effect of LP on cardiac *Fgf23* expression was also observed in FGFR4^−/−^ mice (*p* = 0.0246) (Figure 3B). Further investigation into the expression of genes which are involved in the posttranslational regulation of FGF23 showed that mRNA levels of *Polypeptide N-acetylgalactosaminyltransferase 3* (*Galnt3)*, *Furin*, and *Family with sequence similarity 20 member C golgi associated secretory pathway kinase* (*Fam20c*) were not significantly altered in the heart (Figure 3C) or in bone (Figure 3D) of wild-type mice in LP compared to NP.

Next, we wanted to determine whether pregnancy-associated elevations in serum FGF23 levels result in changes in phosphate metabolism. We observed significant increases in serum concentrations of phosphorus (wild-type *p* = 0.0196; FGFR4^−/−^ *p* < 0.0001) and calcium (wild-type *p* = 0.0001; FGFR4^−/−^ *p* = 0.0026) in wild-type and FGFR4^−/−^ mice during LP when compared to NP mice (Figure 4A,B). Additionally, pregnant FGFR4^−/−^ mice had significantly greater increases in both serum phosphorus (*p* = 0.0093) and serum calcium (*p* = 0.0364) concentrations when compared to pregnant wild-type mice. The renal expression levels of sodium-dependent phosphate co-transporters *Slc34a1* and *Slc34a3* (Figure 4C) and of the FGF23 receptors *Klotho*, *Fgfr1*, and *Fgfr4* (Figure 4D) were not altered in wild-type mice during LP compared to NP, as determined by RTqPCR. Altogether, these findings suggest that in mice during LP, FGF23 is produced by the heart, but not in bone, and that in pregnancy the kidney does not alter the expression of phosphate metabolism-related genes in response to elevated serum levels of FGF23 and phosphorus.

### 3.3. Serum of Fed Pythons Induces the Hypertrophic Growth of Cardiac Myocytes via FGFR4

To investigate a potential role of FGFR4 in another vertebrate model of physiologic hypertrophy, we studied the infrequently feeding Burmese python (*Python bivittatus*). Fed pythons are a model of extreme physiological regulation due to the sudden and severe increase in mass that many organs experience, including a 40% increase in heart mass, after a large meal [36,37,38]. Water snakes (*Nerodia sipedon*) are frequent feeders and do not experience the same postprandial increase in organ mass [43,44] and were used as controls. We treated primary neonatal rat ventricular myocytes (NRVMs) for 48 h with media containing serum from fasted or fed snakes (diluted to 2% of the total media volume) or with recombinant FGF2 or FGF23 (25 ng/mL) (Figure 5). FGF2 served as a positive control for myocyte hypertrophy [34]. NRVMs treated with python serum from twelve hours post-feeding (12HPF; *p* < 0.0001) and three days post-feeding (3DPF; *p* < 0.0001), with FGF2 (*p* = 0.0030) or with FGF23 (*p* < 0.0001), showed significant increases in cell area compared to control NRVMs that were vehicle-treated (Ctrl). In contrast, incubation with either fed or fasted water snake serum did not increase NRVM area. When cells were co-treated with BLU9931, a selective small-molecule inhibitor for FGFR4 [45], the pro-hypertrophic effect of FGF23 (*p* < 0.0001), 12HPF (*p* < 0.0001), and 3DPF (*p* = 0.0003) python serum was abrogated. The area of NRVMs treated with FGF23 (*p* < 0.0001) or 12HPF (*p* = 0.0003) and 3DPF (*p* = 0.0016) serum was significantly greater than the area of myocytes treated with fasted python serum. These findings suggest that serum of fed pythons contains factor(s) that induce the hypertrophic growth of cardiac myocytes in an FGFR4-dependent manner.

## 4. Discussion

Our study shows for the first time that FGFR4 signaling plays an important role in the growth of cardiac myocytes during physiologic cardiac hypertrophy. Additionally, our data indicate that the heart becomes a significant source of FGF23 during pregnancy, suggesting that heart-derived FGF23 might act as a paracrine ligand for cardiac FGFR4 activation. First, we use pregnant FGFR4^−/−^ mice to demonstrate that increased width, or concentric growth, of individual cardiac myocytes in pregnancy-induced physiologic cardiac hypertrophy is dependent on FGFR4, but that this mechanism does not affect the hypertrophic increase in heart mass. Second, we show that *Fgf23* expression is significantly elevated in the heart, but not in bone, of pregnant wild-type mice, and neither organ experiences a significant change in the expression of posttranslational regulators of FGF23. Third, we corroborate previous studies that indicate a reduced responsiveness of the kidney to elevations in serum FGF23 in pregnancy, and we are the first to present a pregnancy model with significant increases in serum concentrations of phosphorus and calcium during late pregnancy. Fourth, we use fed pythons as another vertebrate model of physiologic hypertrophy to demonstrate that the pro-hypertrophic effects of serum on cultured cardiac myocytes are dependent on FGFR4.

We have identified FGFR4 as a receptor tyrosine kinase that induces physiologic cardiac hypertrophy. The signaling mechanisms involved in the propagation of physiologic hypertrophy have been found to involve multiple inducers, including VEGF B and insulin/IGF1, that activate the receptor tyrosine kinases VEGF receptor 1 and IGF1 receptor, respectively [12,13,14,15,16]. These stimuli trigger intracellular signaling pathways specific to physiological hypertrophy, such as the phosphoinositide-3-kinase/Ak strain transforming (PI3K/Akt) pathway, and the extracellular signal-regulated kinase 1/2 (ERK1/2) signaling pathway [46]. Intracellular Ca^2+^ has also been implicated as a signal for cardiac hypertrophy. Intracellular Ca^2+^ concentrations increase in response to myocyte stretching and increased load on working heart preparations [47,48,49]. This increase of intracellular Ca^2+^ activates calcineurin/NFAT signaling, another signaling pathway involved in the progression of physiologic and developmental hypertrophy [50,51,52,53]. Angiotensin II, phenylephrine, and endothelin-1 also elevate intracellular Ca^2+^ concentrations and induce the hypertrophic response in cardiac myocytes [54,55,56,57]. Various FGF isoforms have been shown to regulate cardiac remodeling. Paracrine FGF2 activates MAPK signaling, inducing hypertrophic growth of cardiac myocytes, though the role of FGF2 in physiologic hypertrophy is not known [58,59,60]. FGF16 also plays a role in cardiac hypertrophy, though the role is the opposite of FGF2 in cardiac hypertrophic remodeling. FGF16 and FGF2 compete for the same primary receptor, FGFR1c, which causes increased FGF16 activity to reduce FGF2-induced cardiac remodeling [61,62]. FGF21, an endocrine FGF, has also been found to directly affect cardiac remodeling. Short-term elevations of FGF21 during hypertension, ischemia–reperfusion injury, and β-adrenergic activation have been shown to be cardioprotective [63,64,65,66,67,68,69,70] and prevent pathologic cardiac hypertrophy through the activation of FGFR1c/β-klotho [71,72], promoting antioxidant gene expression and inhibiting the formation of reactive oxygen species [73]. Additionally, elevated FGF21 during type 2 diabetes has been found to activate FGFR4-mediated ERK1/2 signaling and subsequently promote pathological cardiac hypertrophy [74], demonstrating that endocrine FGFs may have vastly different effects in tissues based on the internal milieu of a subject or tissue. We have previously reported that elevations of serum FGF23 can induce cardiac myocyte hypertrophy via FGFR4-mediated PLCγ/calcineurin/NFAT signaling, and that deleting or blocking FGFR4 abrogates the hypertrophic effects observed in mice on high-phosphate diet and in a rat model of CKD [26,34]. Though these studies have focused on pathologic hypertrophy, the significant elevation of FGF23 over the course of exercise training [32] and pregnancy [75] suggests that FGF23/FGFR4 signaling could play a role in physiologic cardiac hypertrophy as well, which is supported by our study.

The near-doubling of serum FGF23 concentrations in our pregnant mice is consistent with a previous publication [75], and pregnant FGFR4^−/−^ mice exhibited similar increases in both intact and total FGF23. Due to the decrease in cardiac *Fgfr1* expression and unchanged *Fgfr4* expression in pregnancy, it is likely that FGF23 primarily signals through FGFR4. Regardless of genotype, pregnant mice develop cardiac hypertrophy, represented via increased heart-weight-to-tibia-length ratios. One morphological difference between pregnant wild-type and FGFR4^−/−^ mice observed in our study is that the individual myocyte cross-sectional area was not increased in FGFR4^−/−^, as it was in wild-type mice. Additionally, FGFR4^−/−^ mice had significantly more connective tissue fractions, regardless of pregnancy status, when compared to wild-type hearts. Though wild-type cardiac myocyte size was increased, there was no observable difference in left ventricular free-wall thickness or septum thickness between pregnant mice and controls in both genotypes, and this aligns with the expectation that physiologic cardiac hypertrophy is predominantly eccentric hypertrophy, characterized by myocyte elongation and increased numbers of sarcomeres per myocyte [76]. Even though the increased cardiac myocyte width in wild-type mice was observed on an individual cell level, the heart morphology was unchanged, consistent with the expected maintenance of normal morphology in physiologic hypertrophy [76]. The increased percentage of connective tissue in the FGFR4^−/−^ hearts compared to wild-type hearts, in the absence of increased myocyte size that is seen in wild-type hearts, may also explain why heart morphology was not significantly different between genotypes.

In pregnant FGFR4^−/−^ mice, individual cardiac myocytes did not experience any increased cell diameter. This can be explained by previous studies that have linked calcineurin/NFAT signaling to both pathologic and physiologic hypertrophy [52] and that have shown that activation of this pathway is involved in cardiac myocyte hypertrophy [77]. In hypertrophy, cardiac myocyte width is increased due to the production of more parallel sarcomeres [3]. Although it is unknown whether FGFR4 signaling dictates sarcomere patterning, decreased parallel patterning of sarcomeres during hypertrophic growth could account for the differences seen between the concentric growth of individual myocytes in pregnant wild-type and FGFR4^−/−^ mice while maintaining hypertrophic growth of the heart overall. We hypothesize that the increase in the heart weight observed in pregnant FGFR4^−/−^ mice is, at least in part, due to an increase in eccentric myocyte growth, with increased production of sarcomeres in sequence, without an increase in concentric growth of myocytes due to increased parallel sarcomere patterning or cytoplasmic volume. Future studies should investigate the possible role of FGFR4 in the determination of sarcomere patterning.

It is possible that the increased collagen content observed in our FGFR4^−/−^ mice is due to the role of FGFR4 signaling in myogenesis. We do not interpret the increases in cardiac collagen staining in FGFR4^−/−^ mice as fibrosis, since fibrosis is defined as the development of fibrous connective tissue as a reparative response to injury or damage, either as part of the normal healing process or in excess as part of pathology. It is possible that the observed increase in connective tissue fraction is a function of development without functional FGFR4 signaling, rather than a response to injury or damage. It has been observed that FGF4, a paracrine member of the FGF family, is involved in the aggregation of cardiomyocytes with cardiac fibroblasts [78], and embryonic *Fgf4* downregulation inhibits myogenesis [79], likely through the involvement of FGFR4 [80]. Further research demonstrated that Pax3 regulation of FGFR4 signaling activation modulates the progression of muscle progenitor cells into the myogenic program [81]. However, FGFR4^−/−^ mice develop normally without evident muscle defects, pointing towards a compensation of other FGFR isoforms that take over the role of FGFR4 in myogenesis [39]. Though muscle myogenesis may not be negatively affected, our data indicate that the lack of FGFR4 is likely decreasing the number of progenitor cells entering the myogenic program during development, leading to an increased number of cells that contribute to the increased connective tissue development in FGFR4^−/−^ hearts. Future studies need to investigate whether cardiac function in FGFR4^−/−^ is different from that in wild-type mice, as would be expected as a consequence of the increases in connective tissue during development. Furthermore, these data indicate a possible role of FGFR4 in the regulation of connective tissue development. Future studies should investigate the effects of FGFR4 deletion on the development of connective tissue in other organs in order to determine whether this effect is exclusive to muscle tissues or if it occurs in other organ tissues as well. Additionally, these data indicate that the blocking of FGFR4 can have a lasting effect on a developing heart, and therefore future therapies targeting FGFR4 should be additionally scrutinized before prescription to patients whose hearts are not fully developed.

FGF23 is primarily produced by the bone, but other organs such as the heart [82], liver [83,84], and kidneys [85,86] have been identified as additional sources of FGF23. To our surprise, we did not detect significant alterations in *Fgf23* expression in bone of pregnant mice. Additionally, pregnant mice did not have increased liver expression of *Fgf23*. Previous studies have shown that healthy kidneys do not produce *Fgf23* to a significant degree [85], and placentas of pregnant mice also do not contribute to FGF23 production [33]. The heart of pregnant mice was the only organ that we studied and found to exhibit increased *Fgf23* expression when compared to non-pregnant mice. The heart has been shown to be a source of FGF23 in models with pathologic hypertrophy [87], supporting the ability of the heart to produce FGF23 in times of cardiovascular enlargement. Post-translational modifiers of FGF23 were not significantly altered in the bone or hearts of pregnant mice, though there was a trended decrease in bone *Galnt3* expression (*p* = 0.0558). Galnt3 is known to protect FGF23 from cleavage [88,89], so decreased expression would lead to a decreased ratio of intact:cleaved FGF23, which indicates that the FGF23 produced in the bone would be increasingly cleaved in pregnant mice.

Our serology results showing significant increases in serum phosphorus and calcium in wild-type and FGFR4^−/−^ pregnant mice differ from previous publications. Previous studies have shown that humans experience a decrease in serum calcium [90,91], and wild-type mice on 1% calcium diets have shown no change in serum calcium concentrations during pregnancy [33,92,93,94]. Furthermore, serum phosphorus concentrations have been shown to remain normal across the duration of pregnancy in humans [90,91,95,96,97,98,99,100,101] and mice [33,93]. Previous mouse studies have primarily used Black Swiss mice or mouse lines with different gene deletions [33,93]. Differences between studies could be due to differences in the genetic background of mouse lines or to measurements being taken after fasting, as we observed the same pattern in two different mouse models from a C57BL/6 background and our mice had 24 h access to food. Additionally, these studies measured serum mineral content using colorimetric assays (Diagnostic Chemicals Limited, Charlottetown, PEI, Canada, [93]; and Sekisui Diagnostics PEI Inc., Charlottetown, Prince Edward Island, Canada, [33]). Our serum calcium and phosphorus concentrations were measured by different colorimetric assays at IDEXX and UNC, which both provided consistent measurements.

In our current study, the ratio of intact-to-total FGF23 was not significantly different between pregnant mice and controls in either genotype. In pathologies accompanied by increased FGF23, the ratio of intact and cleaved FGF23 is altered. In CKD, dialysis patients have significantly increased FGF23, with most of it being intact, biologically active protein [102]. Iron-deficiency-induced increases in FGF23 are accompanied by increased FGF23 cleavage, resulting in marginal to no increase in intact FGF23, with higher total FGF23 and reducing the ability of FGF23 to perform its phosphaturic functions in the kidney and parathyroid [103,104]. Previous studies have reported intact-to-total FGF23 ratios showing ~65% and ~68% intact FGF23 in healthy adult C57BL/6 mice [105], ~72% intact FGF23 in furin-deleted mice on normal phosphorus diet [106], and as low as ~40% intact FGF23 in wild-type mice [107]. In our studies, pregnant and non-pregnant wild-type mice exhibited serum FGF23 levels with 68% intact FGF23, similar to the control mice of previous studies. Non-pregnant and pregnant FGFR4^−/−^ mice had higher ratios of intact to total FGF23 (71% and 83%, respectively), but the increases were not significantly different between pregnancy status (*p* = 0.1330) or versus non-pregnant wild-type mice (*p* = 0.1469). The “normal” ratio of functional to total FGF23 was preserved in our experiments, showing that the pregnant mouse is not compensating for the increase in FGF23 by reducing its functionality through increased cleavage, as in anemia, or by reducing FGF23 cleavage, as in CKD. This finding indicates that the increased FGF23 in pregnancy should be capable of signaling in the kidney and parathyroid, as it would be in a healthy non-pregnant mouse. However, an interesting aspect of our study is that in pregnancy, serum levels of intact FGF23 are elevated, while physiologic targets of FGF23 in the kidney do not undergo alterations, at least not on the mRNA level.

It has previously been shown that phosphate filtration and resorption are unaltered during pregnancy in humans [108] and urine phosphorus does not significantly change during pregnancy in mice, despite elevated FGF23 [33,93]. Additionally, previous research has shown that elevated intact serum FGF23 in pregnant *Phex*-null mice is unable to blunt the pregnancy-related increase in calcitriol [33]. The uniquely significant elevations in serum concentrations of phosphorus and calcium in our pregnant mice studies suggest that a phosphaturic response would be activated in the kidneys of these mice. We observed similar unresponsiveness in the expression of genes in pregnant kidneys. Renal *klotho* expression is responsive to FGF23 elevations, showing decreased expression in mice administered injections of recombinant FGF23 without further intervention [109], but we observed no change in *klotho* expression in pregnant mice with two-fold elevations of FGF23. Additionally, the high levels of intact FGF23 did not decrease renal expression of *Slc34a1* and *Slc34a3* or reduce serum concentrations of phosphorus, which is the primary physiological function of intact FGF23. FGFR1 was present in the kidney during pregnancy, indicating that the receptor complex necessary for FGF23 function is available, but the kidney is not responding to the elevations of intact FGF23 in the blood in the way healthy non-pregnant mice respond. One possible, but currently untested, hypothesis for how this may work is that the FGF23 protein produced in the hearts of pregnant mammals could have different post-translational modifications that inhibit FGF23’s interaction with FGFR1/klotho or promote increased paracrine signaling, making a fraction of the increased FGF23 unable to function normally in the kidney and parathyroid. Alternatively, there may be factors increased during pregnancy that bind FGF23 before or after secretion into the blood of pregnant mammals that prevents filtration of FGF23 by the kidney. Future studies need to determine whether the kidney is indeed resistant to elevations of heart-derived, intact FGF23 during pregnancy.

Our findings in mice were further supported by experiments using fed Burmese pythons (*Python bivittatus*), another vertebrate model of physiologic cardiac hypertrophy. The infrequently feeding Burmese python has been described as a model of extreme physiological regulation due to the sudden and severe increase in mass that many organs experience, including a 40% increase in heart mass, after a large meal [36,37,38]. Previous studies have added plasma from fed pythons to NRVM cultures and detected an induction of hypertrophic growth; however, the specific stimuli causing this effect have yet to be identified [40]. NRVMs treated with fed python serum drawn at timepoints when the python was experiencing hypertrophic organ growth showed a significant increase in cell area that occurred in an FGFR4-dependent manner, further demonstrating the role of FGFR4 in myocyte growth during states of physiologic hypertrophy. BLU9931 is a highly selective and irreversible small-molecule inhibitor of FGFR4 [45] that covalently binds Cys552 within the ATP-binding pocket of FGFR4, which is not present in FGFR1-3 [110]. This makes it unlikely that we are mistaking the inhibition of another receptor as inhibition of FGFR4 in these experiments. FGF23 treatments and treatments with fed python serum caused a similar increase in myocyte size and were similarly abrogated by co-treatment with BLU9931 (Figure 5). Though this finding does not definitively show that FGF23 is a circulating, pro-hypertrophic factor in pythons, it does imply that FGF23 is a likely candidate for inducing hypertrophic growth in NRVMs treated with serum from fed pythons. Further studies are needed to identify the specific ligand responsible for FGFR4 activation in fed python serum, but the lack of species-specific antibodies and genome analysis presents obstacles. Though the *P. bivittatus* genome contains an ortholog of FGF23 (NCBI Gene ID: 103051058), our group was unable to measure *P. bivittatus* FGF23 by using antibodies against human, mouse, and rat FGF23.

Future studies will need to determine whether FGF23 is the responsible ligand for FGFR4 activation during physiologic hypertrophy, though our data and previously published studies strongly indicate that FGF23 is the most likely culprit. As we previously discussed, paracrine FGF2 signaling can induce hypertrophic growth of cardiac myocytes [58,59,60], but when we treated NRVMs with FGF2, the hypertrophic effects were less than that caused by fed python serum, and the inhibition of FGFR4 did not have as extreme of an inhibitory effect on FGF2-induced cardiac myocyte growth. This leads us to believe that the effect observed in NRVMs treated with fed python serum is not primarily caused by FGF2 interactions. FGF21 also has the ability to influence cardiac hypertrophy [74], but it is known that FGF21 levels decrease significantly during the first two trimesters of pregnancy [111], while cardiac output begins to increase in the first trimester and continues to do so into the third trimester [112,113]. This makes it very unlikely that FGF21 is playing a role in this process. It will be important to conduct pregnancy studies in mice with cardiac-specific FGF23 deletions to determine whether the heart is the primary source of increased FGF23 in pregnancy and whether heart-derived FGF23 is responsible for the increased area of individual myocyte observed in pregnant wild-type mice.

## 5. Conclusions

Our study demonstrates the contribution of FGFR4 to the concentric growth of individual cardiac myocytes during physiologic hypertrophy, and we postulate that in this context, FGF23 is a candidate ligand for FGFR4 activation. We uncover pregnancy as a physiologic scenario where the heart produces FGF23 to increase cardiac myocyte size while the kidney does not respond to FGF23 elevations with increases in phosphate excretion. Thus, the development of physiologic cardiac hypertrophy can occur in the absence of renal phosphate wasting. Combined with previous studies, our analyses highlight the importance of FGFR4 signaling in all forms of cardiac hypertrophy.

## Figures and Tables

**Table 1:** Mouse primer sequences. The following oligonucleotides shown in 5′ to 3′ orientation were used as primers in quantitative real-time PCR analyses.

Gene	Orientation	Primer Sequence (5′ to 3′)
*Fgf23*	Forward	CAC TGC TAG AGC CTA TCC
	Reverse	CAC TGT AGA TGG TCT GAT GG
*Fgfr1*	Forward	CAA CCG TGT GAC CAA AGT GG
	Reverse	TCC GAC AGG TCC TTC TCC G
*Fgfr4*	Forward	TGA AGA GTA CCT TGA CCT CCG
	Reverse	TCA TGT CGT CTG CGA GTC AG
*Klotho*	Forward	TGT ATG TGA CAG CCA ATG GAA TCG
	Reverse	GAA TAC GCA AAG TAG CCA CAA AGG
*Galnt3*	Forward	ACA CTA TTT ACC CGG AAG CG
	Reverse	AGC TCC TTC TGG ATG TTG TG
*Furin*	Forward	AGC GGC AAC CAG AAT GAG AA
	Reverse	AGG TTC TTG TTG GCC TCC AG
*Fam20c*	Forward	GCC AAG TTG TTT GAG CAC CC
	Reverse	GCT TTT GTC CCC GTG ACA GT
*Slc34a1*	Forward	TCA TTG TCA GCA TGG TCT CCT C
	Reverse	CCT GCA AAA GCC CGC CTG
*Slc34a3*	Forward	GAT GCC TTT GAC CTG GTG GA
	Reverse	GCC ATG CCA ACC TCT TTC AG
*Gapdh*	Forward	CCA ATG TGT CCG TCG TGG ATC T
	Reverse	GTT GAA GTC GCA GGA GAC AAC C

## Data Availability

The original contributions presented in the study are included in the article, further inquiries can be directed to the corresponding authors.
